# Large-scale proximity extension assay reveals CSF midkine and DOPA decarboxylase as supportive diagnostic biomarkers for Parkinson’s disease

**DOI:** 10.1186/s40035-023-00374-w

**Published:** 2023-09-04

**Authors:** Wojciech Paslawski, Shervin Khosousi, Ellen Hertz, Ioanna Markaki, Adam Boxer, Per Svenningsson

**Affiliations:** 1 Laboratory of Translational Neuropharmacology, Department of Clinical Neuroscience, Karolinska Institutet, Stockholm, Sweden; 2 Memory and Aging Center, University of California, San Francisco, San Francisco, CA USA; 3 Basic and Clinical Neuroscience, Institute of Psychiatry, Psychology and Neuroscience, King’s College London, London, UK; 4 Old Age Psychiatry, Institute of Psychiatry, Psychology and Neuroscience, King’s College London, London, UK

**Keywords:** Atypical Parkinsonian disorders, Biomarker, Cerebrospinal fluid, Corticobasal syndrome, DOPA decarboxylase, Midkine, Multiple system atrophy, Parkinson’s disease, Progressive supranuclear palsy, Proximity extension assay

## Abstract

**Background:**

There is a need for biomarkers to support an accurate diagnosis of Parkinson’s disease (PD). Cerebrospinal fluid (CSF) has been a successful biofluid for finding neurodegenerative biomarkers, and modern highly sensitive multiplexing methods offer the possibility to perform discovery studies. Using a large-scale multiplex proximity extension assay (PEA) approach, we aimed to discover novel diagnostic protein biomarkers allowing accurate discrimination of PD from both controls and atypical Parkinsonian disorders (APD).

**Methods:**

CSF from patients with PD, corticobasal syndrome (CBS), progressive supranuclear palsy (PSP), multiple system atrophy and controls, were analysed with Olink PEA panels. Three cohorts were used in this study, comprising 192, 88 and 36 cases, respectively. All samples were run on the Cardiovascular II, Oncology II and Metabolism PEA panels.

**Results:**

Our analysis revealed that 26 and 39 proteins were differentially expressed in the CSF of test and validation PD cohorts, respectively, compared to controls. Among them, 6 proteins were changed in both cohorts. Midkine (MK) was increased in PD with the strongest effect size and results were validated with ELISA. Another most increased protein in PD, DOPA decarboxylase (DDC), which catalyses the decarboxylation of DOPA (*L*-3,4-dihydroxyphenylalanine) to dopamine, was strongly correlated with dopaminergic treatment. Moreover, Kallikrein 10 was specifically changed in APD compared with both PD and controls, but unchanged between PD and controls. Wnt inhibitory factor 1 was consistently downregulated in CBS and PSP patients in two independent cohorts.

**Conclusions:**

Using the large-scale PEA approach, we have identified potential novel PD diagnostic biomarkers, most notably MK and DDC, in the CSF of PD patients.

**Supplementary Information:**

The online version contains supplementary material available at 10.1186/s40035-023-00374-w.

## Background

Parkinson’s disease (PD) diagnosis is based on clinical criteria, and there is a need of biomarkers that reflect ongoing brain pathology. Earlier attempts to find biofluid biomarkers in PD have mostly been hypothesis-driven, quantifying individual markers for known pathological mechanisms. A few untargeted proteomic studies have attempted to find cerebrospinal fluid (CSF) biomarkers with an unbiased approach [1, 2]. However, there are often issues with reproducibility or validation of candidates using orthogonal methods and many proteomic studies tend to lack either sufficient sensitivity or power. However, recently, impressive progress has been made with seed amplification assay, where α-synuclein monomers added to PD CSF induce in vitro aggregation with high diagnostic accuracy [3–5].

PD shares symptomatology with atypical Parkinsonian disorders (APD), including multiple system atrophy (MSA), progressive supranuclear palsy (PSP) and corticobasal syndrome (CBS), which is named corticobasal degeneration (CBD) when pathologically confirmed. An accurate discrimination between PD and APD based on clinical symptoms and signs is often difficult, particularly at early disease stages, but is important as they are different in progression, functional decline, and underlying pathology [6]. CSF or plasma levels of neurofilament light (NfL) differ between PD and APD and can be used to distinguish PD from APD [7–9]. However, the NfL changes are not disease-specific [8] and NfL level is also increased in many other neurodegenerative disorders [9, 10].

Proximity extension assay (PEA) is a highly sensitive 96-plex immunoassay for detecting proteins in biological fluids, employing distinctive antibody–oligonucleotide protein binding for quantitative real-time polymerase chain reaction-based measurement [11]. It bypasses many technical issues that would be seen when translating proteomic findings with immunoassays, as it is already an antibody-based assay, and the samples are not heavily pre-processed before analysis. PEA has previously been used to explore markers for disorders such as AD [12], multiple sclerosis [13] and other dementias [14]. For PD and APD, the PEA assay has been applied using Neurology and Inflammation panels [15, 16].

In the present study, we expanded the search for protein biomarkers of PD and APD using PEA, with a focus on PD. In an unpublished pilot CSF study with PD and control samples against 12 different Olink PEA panels, the Oncology II, Cardiovascular II and Metabolism panels were identified as the most promising. Therefore, all PD, APD and control samples were run on these three panels.

## Materials and methods

### Patient cohorts

The Stockholm patients were followed by a movement disorder specialist and fulfilled the clinical diagnosis criteria for PD [17], MSA [18], PSP [19] or CBS [20]. Patients with any other serious neurological or psychiatric disorder or cancer were excluded from the Stockholm cohort. Control subjects were healthy volunteers or patients for whom investigations had not resulted in any severe neurological diagnosis (e.g., tension headache or sensory symptoms). CSF samples were collected by a movement disorder specialist from patients and controls who underwent lumbar punctures in neurological clinics within Region Stockholm. All study individuals had given written consent to the storage of their samples for future use in studies, and the study was approved by the Swedish Ethical Review Authority (dnr 2020-03684). The PD patients included were additionally part of the Stockholm BioPark cohort (dnr 2019-04967) [21], and were clinically assessed by a movement disorder specialist. The disease severity of the PD patients was evaluated using the Unified Parkinson's disease rating scale [22] and Hoehn & Yahr scale, and their cognition assessed using the Montreal Cognitive Assessment scale [23]. PD medications are summarized as *L*-dopa equivalent daily dose (LEDD) [24] and *L*-dopa dose (LDD). Additionally, the patients completed self-report questionnaires including Parkinson’s Disease Questionnaire 39 [25], Hospital Anxiety and Depression Scale [26], Non-Motor Symptoms Questionnaire [27], and Pittsburgh Sleep Quality Index [28].

The PD validation cohort used in the study originated from BioFIND (http://biofind.loni.usc.edu), an observational, multi-center, cross-sectional study of moderate-to-advanced PD participants [29]. Enrolled PD participants met the modified United Kingdom PD Society Brain Bank (UKPDBB) clinical diagnostic criteria that require all three classic motor signs of PD (tremor, bradykinesia, and rigidity) to be present for study enrolment. For the BioFIND cohort, PD patients did not have any other serious neurological or psychiatric disorder, a history of any major medical condition (e.g., cancer, liver disease, autoimmune disorders, or hematological disorders), early-onset autonomic symptoms, prior brain surgery (including for DBS placement), or any use of investigational drugs. The institutional review board of BioFIND approved the study protocol. Written informed consent was obtained from each study participant.

A smaller validation cohort of controls along with pathologically confirmed PSP and CBD patients from University of California, San Francisco (UCSF) was used as a validation cohort set. Details on demographics of the used cohorts are presented in Additional file 1: Tables S1, S2 and S3.

### Chemicals

Unless stated otherwise, all chemicals were purchased from Sigma-Aldrich (Merck KGaA, Darmstadt, Germany) and were of analytical grade. All solutions were prepared using Milli-Q deionized water (Millipore, Burlington, MA).

### Sample collection

For BioPark and UCSF samples, standardized lumbar puncture was performed as described before [30, 31]. Briefly, CSF collection was performed in a sitting-up bending-forward position, at the L3–L4 interspace, in accordance with the Alzheimer’s disease Neuroimaging Initiative recommended protocol. Samples were collected into sterile polypropylene tubes. After discarding the first 2 ml, 10–12 ml of CSF sample was collected and gently mixed in order to minimize the gradient influence. Cell counts were measured and samples were centrifuged in the original tube at 4000 rpm for 10 min at 4 °C. CSF samples were aliquoted, frozen on dry ice and stored at − 80 °C until analysis. The time between sample collection and freezing was not greater than 30 min. Blood-contaminated samples were excluded (erythrocyte count > 10 cells/ml). For BioFIND samples, standardized lumbar puncture was performed at L3–L4 or L4–L5 interspace as described before [29]. There was no statistical difference in the collection site between controls and PD cases (*P* = 0.41).

### PEA

Multiplex PEA was performed using the Olink platform. All samples were simultaneously run on three panels, Oncology II, Cardiovascular II and Metabolism. The list of proteins included in each panel and the Olink panel validation data are freely available online (https://www.olink.com/data-you-can-trust/validation/). The biological functions of proteins of interest were obtained from the UniProt database (www.uniprot.org) and their tissue expression profiles were assessed using data available on the GTEx database (www.gtexportal.org). The data for each protein are given as a normalised protein expression (NPX) value, an arbitrary unit on a Log2 scale and normalised to minimise both intra-assay and inter-assay variations.

### Enzyme-linked immunosorbent assay (ELISA)

ELISA was performed using the Human Midkine ELISA Kit (ab193761, Abcam, Cambridge, UK) according to the manufacturer’s instructions. Standards, in triplicates, and samples, in duplicates, were transferred to appropriate wells and the antibody mix was added to each well. The assay’s lower limits of detection and quantification were 14.4 pg/ml and 39.1 pg/ml, respectively. Samples were not diluted for the assay and all fell within the quantifiable range (min: 42.7 pg/ml; max: 1346.57 pg/ml; median: 162.04 pg/ml; 1st quartile: 63.22 pg/ml; 3rd quartile: 369.83 pg/ml). The plate was sealed and incubated for 1 h at room temperature on a plate shaker. Next, each well was washed 3 times in wash buffer and 3,3′,5,5′-tetramethylbenzidine development solution was added to each well. The plate was incubated for 10 min in the dark on a plate shaker and stop solution was added to each well. The optical density was read at 450 nm.

### Statistical analyses

The statistical analyses were performed using RStudio (R version 4.2.1) and GraphPad Prism v7. Proteins with > 30% values falling under the limit of detection (LOD) were excluded from analyses. Density plots were used to assess the distribution of the data. The NPX value for each analyte was corrected for total protein abundance. Group comparisons were performed using ANOVA or *t*-tests after correcting for age and sex with a linear model, and adding residuals on the mean (the vertical distance between a data point and the regression line obtained from the linear model correcting for age and sex). Differential protein expression and volcano plots were made fitting robust linear models with the R Limma package, with diagnosis, age and sex as explanatory variables, and *P*-values were adjusted using the Benjamini–Hochberg method. Receiver operating characteristic (ROC) curve analyses were performed to assess the biomarker potential of proteins of interest for Parkinsonian disorders as well as their performance in differential diagnosis. In subjects with PD, the relationship between levels of significantly changed proteins and clinical scores was assessed using the Spearman correlation test. *P*-values < 0.05 were considered as statistically significant. All figures were made in RStudio (R version 4.2.1). T-distributed Stochastic Neighbour Embedding (t-SNE) plots were created with the Rtsne package, forest plots with the forestplot package, volcano plots with the EnhancedVolcano package, ROC curves with the pRoc package, correlation plots with the corrplot package, and all other figures with the ggplot2 package.

## Results

### Patients’ demographics and data quality

A total of 132 individuals with PD, 67 with APD (21 PSP, 22 CBS, and 24 MSA) and 117 controls were recruited from 3 cohorts: BioPark (Stockholm), BioFIND (USA) and UCSF (Additional file 1: Tables S1, S2 and S3). All CSF samples underwent PEA analysis for 276 proteins (92 for each of the three panels: Oncology II, Cardiovascular II and Metabolism). The selection of the three panels was based on a pilot experiment in 5 PD and 5 control cases using 12 Olink panels (data not shown), of which the top three panels with regard to the number of differentially expressed proteins in PD were selected.

Proteins were excluded if > 30% of samples fell under the LOD, leaving in total 205 proteins to be included in further analyses. The protein levels were normalized to the total protein content of each sample, which corrected for variations related to patients' height, lumbar puncture level, or other technical factors (Additional file 1: Fig. S1). Age and sex were included as covariates in all analyses, either as explanatory variables in linear models, or using residuals following linear model correction. Density plots for individual proteins showed that they were all approximately normally distributed, and boxplots for individual-sample total protein expression verified successful pre-processing and normalisation by Olink. PCAs were plotted to explore potentially confounding covariates, and a systematic shift in the data between the Stockholm and UCSF cohorts was noted, even when accounting for diagnosis, age, and sex. To eliminate any cohort effect, the UCSF samples (11 controls, 10 PSP, 15 CBD) were excluded in the initial analysis and used as a validation set instead.

### Significantly changed proteins in PD

**Fig. 1:**
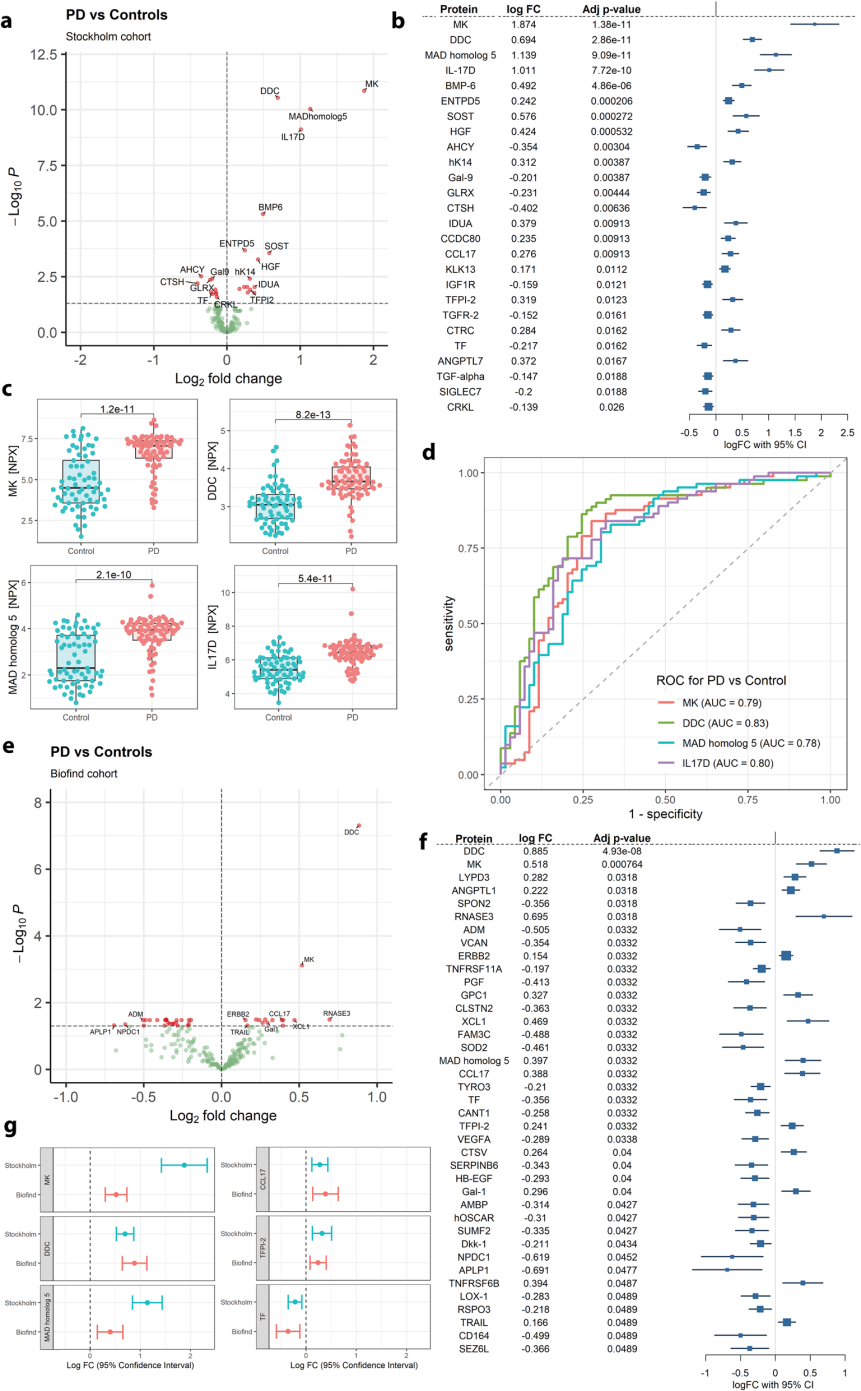
Altered CSF proteins between Parkinson’s disease (PD) and Controls. **a** Volcano plot showing log2 fold change and -log10 adjusted *P*-value in the Stockholm cohort (*n* = 69 for controls; *n* = 81 for PD). **b** Forest plot displaying all significant proteins with log fold change and 95% confidence interval ordered in ascending adjusted *P*-values in the Stockholm cohort. **c** Boxplots of the top 4 most significant proteins in the Stockholm cohort, adjusted for age and sex; levels representing normalised protein expression (NPX) and *P*-values displayed. *T*-test for groupwise comparisons (*n* = 69 for controls; *n* = 81 for PD). **d** Receiver operating characteristic (ROC) curves for the top 4 proteins in the Stockholm cohort with areas under curve (AUC). **e** Volcano plot showing log2 fold change and -log10-adjusted *P*-value in the BioFIND cohort (*n* = 37 for controls; *n* = 51 for PD). **f** Forest plot displaying all significant proteins with log fold change and 95% confidence interval ordered in ascending adjusted *P*-values in the BioFIND cohort. **g** A forest plot comparing overlapping significant proteins

Group fold-changes of NPX values between PD and controls from the BioPark cohort and Benjamini–Hochberg (BH) adjusted *P*-values were used to construct volcano plots to highlight significant markers (Fig. 1a, *P* < 0.05). Twenty-six proteins were identified to be significantly changed and are listed in the forest plot (Fig. 1b) with log fold changes and 95% confidence intervals ordered in ascending *P*-values. The four most significantly changed proteins (Fig. 1c) were midkine (MK), DOPA decarboxylase (DDC), interleukin-17D (IL17D) and mothers against decapentaplegic homolog 5 (MAD homolog 5), which were all elevated in PD compared with controls. ROC curves were constructed to assess their performance in differentiating PD from controls (Fig. 1d), and the area under curve (AUC) was calculated. After correcting for age and sex as covariates, DDC showed the most accurate diagnostic potential (AUC = 0.80) for distinguishing PD from controls, followed by MK (AUC = 0.78), MAD homolog 5 (AUC = 0.75), and IL17D (AUC = 0.74). Since clinical PD diagnosis can occasionally be incorrect at postmortem investigation [32], we further compared the significantly changed CSF proteins between DaTSCAN [33] (i.e., ^123^I ioflupan)-confirmed PD cases (*n* = 52 out of 81) and DaTSCAN-unconfirmed cases from the Stockholm cohort. We found that all of the significantly changed CSF proteins presented in Fig. 1a displayed very similar fold changes and average expression levels between the DaTSCAN-confirmed and unconfirmed cases (Additional file 1: Table S4). Despite the smaller sample sizes and lower power, most of these CSF proteins still showed significant, or close to significant changes (*P* < 0.1) (Additional file 1: Table S4).

### Validation of PD biomarkers in a second cohort

Next, we analysed CSF from the BioFIND cohort individuals using the same approach as for the Stockholm cohort. The group fold-changes of NPX values between PD and controls from the BioFIND cohort and BH-adjusted *P*-values were used to construct volcano plots to highlight significant markers (Fig. 1e, *P* < 0.05). Thirty-nine significantly changed proteins were identified and are listed in a forest plot (Fig. 1f) ordered in ascending *P*-values. Similar as that found in the Stockholm cohort, MK and DDC were the most changed proteins in PD compared to controls, and significant changes were also observed for SMAD5, C–C motif chemokine 17 (CCL17), tissue factor pathway inhibitor 2 (TFPI-2), and tissue factor (TF) with similar trends of difference (Fig. 1g).

### DDC correlates with levodopa treatment in PD patients

**Fig. 2:**
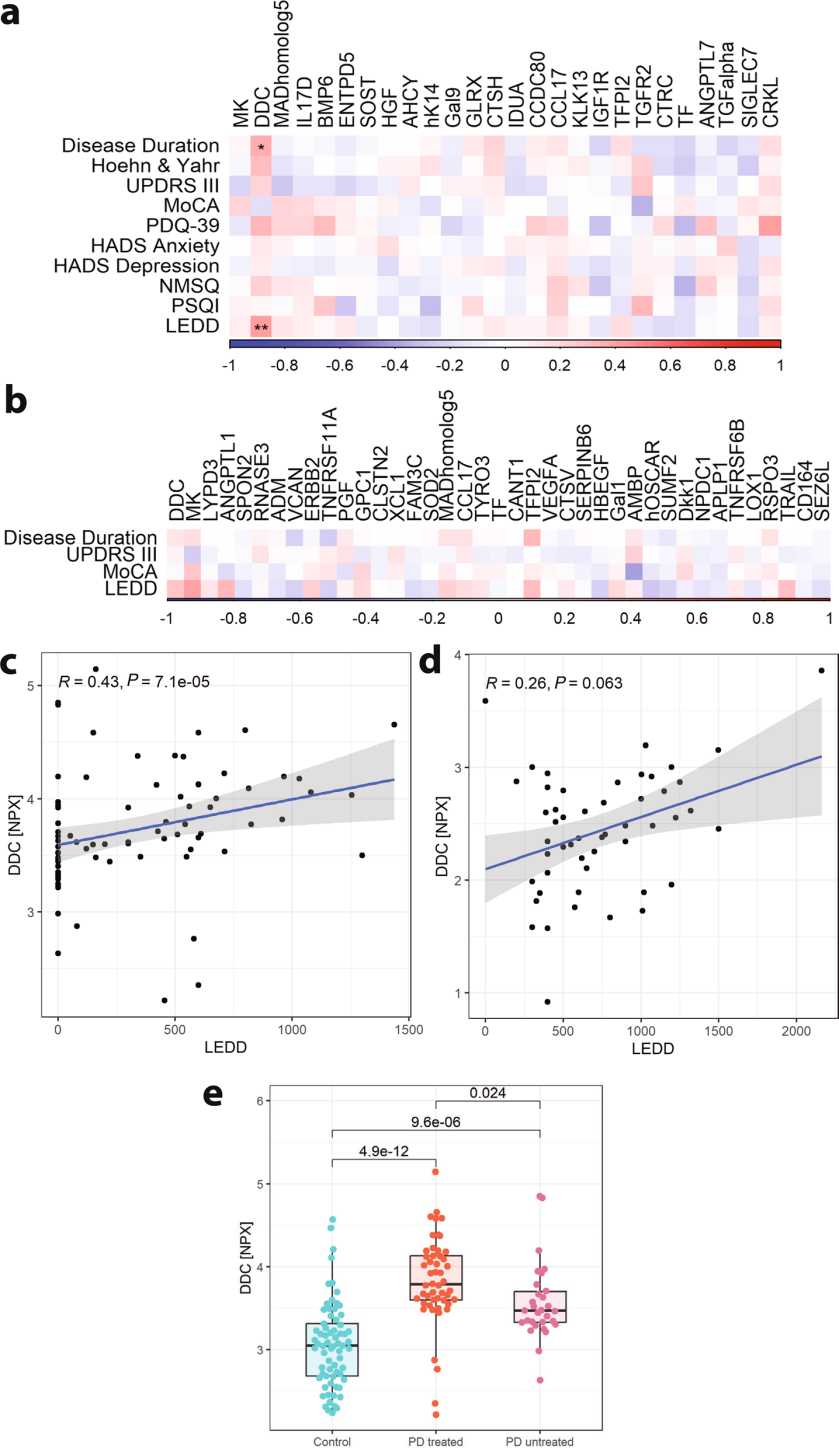
Association of CSF protein levels with clinical parameters of Parkinson’s disease (PD) patients. **a** Spearman’s rank, adjusted for age and sex, between CSF levels of significantly changed proteins in PD patients from the Stockholm cohort and disease duration, scores of Hoehn&Yahr, Unified Parkinson’s Disease Rating Scale (UPDRS) part 3, Montreal Cognitive Assessment (MoCA), Hospital Anxiety and Depression Scale (HADS), Non-Motor Symptoms Questionnaire (NMSQ) and Pittsburgh Sleep Quality Index (PSQI), and Levodopa equivalent daily dose (LEDD) (BH adjusted *P*-values; *P < 0.05; **P < 0.01). **b** Spearman’s rank, adjusted for age and sex, between CSF levels of significantly changed proteins in PD patients from the BioFIND cohort and disease duration, scores of UPDRS part 3 and MoCA, and LEDD (BH-adjusted *P*-values). **c**, **d** Correlation of LEDD with aromatic *L*-amino-acid decarboxylase levels (DDC) in the Stockholm (**c**) and BioFIND (**d**) cohorts (Spearman’s ρ, adjusted for age and sex). **e** Group comparisons (*t*-test; *P*-values displayed, adjusted for age and sex) between controls and PD with or without medication (PD treated and PD untreated, respectively)

Next, we investigated whether any of the significantly differentiating proteins correlated with clinical parameters of PD patients. The PD patients from the BioPark cohort [21] were assessed for motor symptoms, cognition, disease duration and LEDD, and self-assessed for several non-motor symptoms including sleep, anxiety and depression. We investigated associations between clinical parameters and CSF levels of the 26 significantly changed proteins in PD using Spearman’s rank, and after correcting for multiple comparisons, only correlations of DDC with disease duration and LEDD remained significant (Fig. 2a). No significant correlations were observed for the BioFIND cohort (Fig. 2b). As DDC showed a strong correlation with LEDD in the Stockholm cohort (Fig. 2c, Spearman’s rank, *P* = 7.1 × 10^–5^) and a trend of correlation in the BioFIND cohort (Fig. 2d, Spearman’s rank, *P* = 0.063), we compared DDC levels in controls versus PD patients with or without anti-Parkinsonian treatment (i.e., LEDD). We used only the Stockholm cohort, since only one untreated patient was available in the BioFIND cohort. We found that DDC values were elevated in both drug naïve (*P* = 9.6 × 10^–6^) and treated (*P* = 4.9 × 10^–12^) PD patients compared to controls (Fig. 2e). DDC was significantly more (*P* = 0.024) elevated in PD patients on anti-Parkinsonian treatment (Fig. 2e). Since levodopa is always administered with DDC inhibitors, we also examined if LDD per se correlated with CSF DDC levels. The CSF DDC was significantly higher both in patients treated with levodopa (*P* = 7.8 × 10^–7^, *P* = 1.4 × 10^–8^ for the Stockholm and BioFIND cohorts, respectively) and untreated with levodopa (*P* = 3.1 × 10^–10^, *P* = 0.031 for the Stockholm and BioFIND cohorts, respectively) compared to controls (Additional file 1: Fig. S2a, b). The correlation between levodopa dose and CSF DDC level was significant only in the Stockholm cohort (Additional file 1: Fig. S2c, d, Spearman’s rank = 0.29, *P* = 0.0094).

Next, we examined if the biomarker protein levels are affected by any other medications (Additional file 1: Table S5), regardless of the disease state. We analysed the effect of a drug type if at least 6 cases of the analysed cohort were under its treatment (Additional file 1: Figs. S3 and S4). For the Stockholm cohort, we observed that the level of Gal1 was affected by treatment with MAO inhibitors (Additional file 1: Fig. S3b). The levels of FS (Follistatin), Erb-B2 receptor tyrosine kinase 2 (ERBB2), TGFR2 (transforming growth factor beta receptor 2), MSLN (mesothelin) and TM (thrombomodulin) were influenced by Benzodiazepines (Additional file 1: Fig. S3c) and PRS27 (serine protease 27) by allergy drugs (Additional file 1: Fig. S3i). Additionally, the levels of RSPO3 (R-Spondin 3), ROR1 (receptor tyrosine kinase like orphan receptor 1) and CDH2 (cadherin 2) were affected by antihypertensive and diuretic medications (Additional file 1: Fig. S3p). Other types of medication did not have significant effect on the analysed proteins in the Stockholm cohort. In the BioFIND cohort, the only significant effects were observed for Amantadine treatment on SUMF2 (sulfatase modifying factor 2) (Additional file 1: Fig. S4c), antidepressants on LPL (lipoprotein lipase) (Additional file 1: Fig. S4e), and painkillers on T-cell surface glycoprotein CD4 (Additional file 1: Fig. S4s).

### Altered levels of several CSF proteins in PD versus APD

**Fig. 3:**
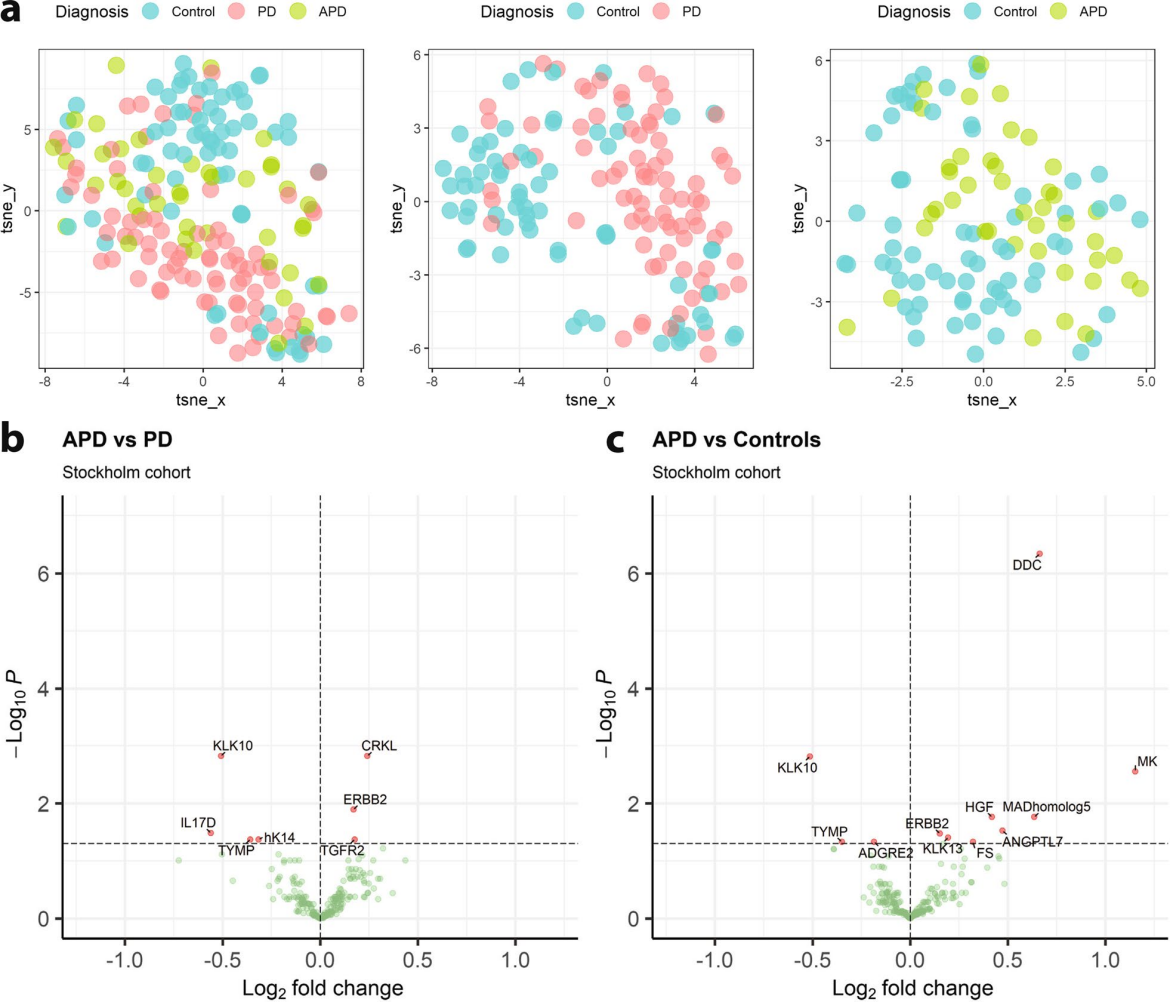
Comparison of CSF proteins between Parkinson’s disease (PD), atypical Parkinsonian disorders (APD) and Controls. **a** t-distributed Stochastic Neighbour Embedding (t-SNE) plots of PD, APD and Controls. **b**, **c** Volcano plots showing log2 fold change and -log10 of adjusted *P*-value for differential protein expression between APD and PD (**b**) and between APD and Controls (**c**)

We further explored whether PD could be differentiated from APD and controls. In order to gain statistical power, we combined 11 PSP, 7 CBS and 24 MSA patients into one APD group to compare with PD patients and controls. As an initial exploratory step we constructed t-SNE plots to visualise the analysed proteome in a two-dimensional manner, and investigated separation according to diagnosis. After adjusting for age and sex, PD appeared to separate from controls with APD falling in the middle (Fig. 3a). For a clearer differential visualisation, controls were plotted with PD and APD separately, displaying a more pronounced diagnostic separation. This suggests that there is not only a general proteomic difference between PD and controls, but also between controls and APD, even though no apparent clusters were visible in the latter.

Subsequently, we plotted volcano plots for APD versus PD (Fig. 3b) where we found 7 significantly altered proteins, and APD versus controls where 11 proteins were significantly changed (Fig. 3c). Interestingly, some of the proteins that were increased in PD compared with controls, were also increased in APD compared with controls (e.g., MK, DDC and MAD homolog 5). Moreover, some proteins, such as Kallikrein Related Peptidase 10 (KLK10), was only changed in APD.

**Fig. 4:**
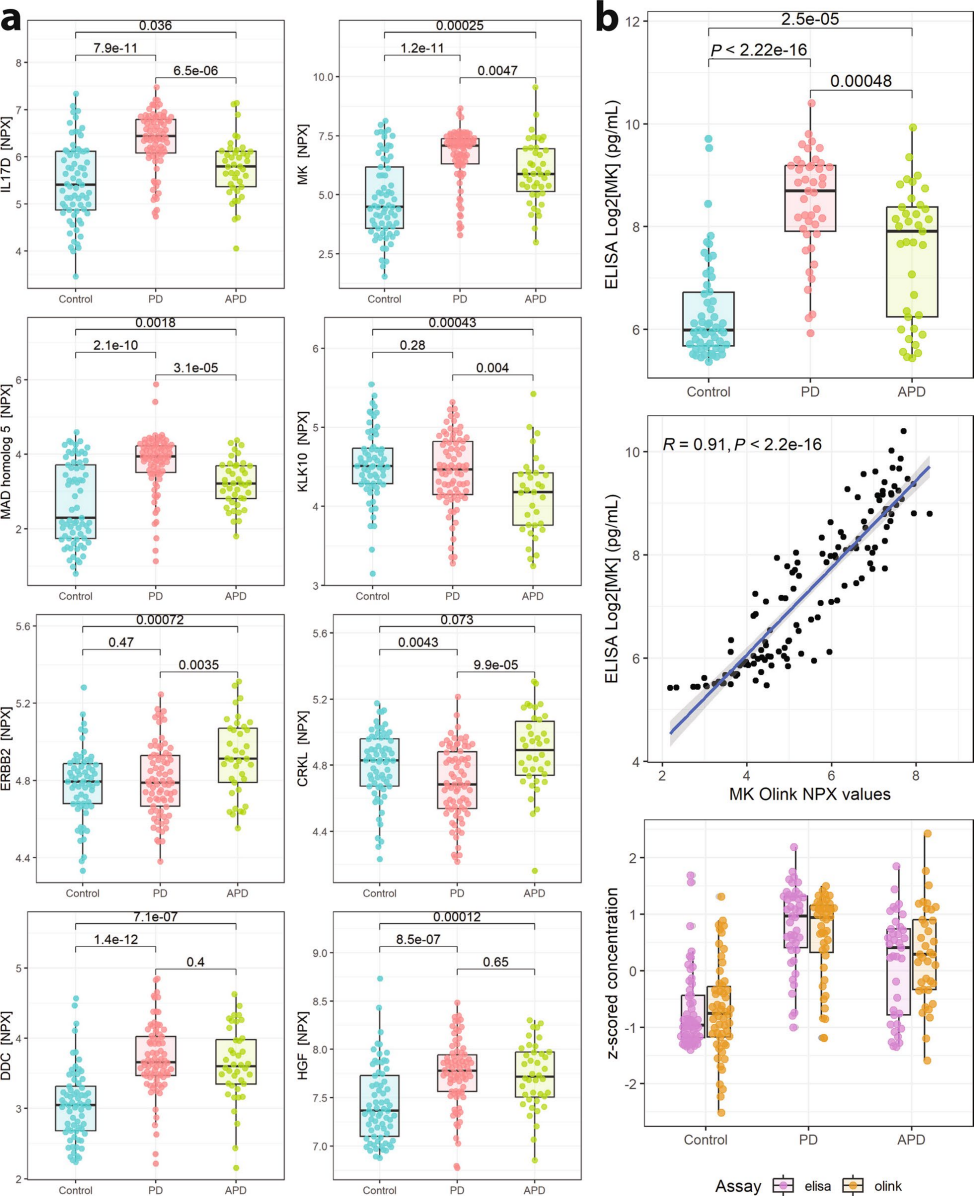
Comparison of CSF proteins between Parkinson’s disease (PD), atypical Parkinsonian disorders (APD) and Controls. **a** Boxplots of the most significantly changed proteins, adjusted for age and sex. Levels are normalised protein expression (NPX). *T*-test for groupwise comparisons. **b** Validation of Midkine (MK) levels using a commercial ELISA assay (upper) and comparison with Olink NPX values by a linear model regression (middle) and z-scored boxplot comparisons (bottom)

Hence, we compared some of these most significantly altered proteins (MK, MAD homolog5, IL17D, ERBB2, KLK10, CRKL, DDC and HGF) between all three groups (Fig. 4a). MK, IL17D and MAD homolog 5 were significantly changed for all pairwise comparisons between PD, APD and controls. DDC and HGF were increased in PD and APD compared to controls. CRKL was lower in PD compared with APD and controls, but was not different between controls and APD. Finally, KLK10 was decreased in APD compared with PD and controls, and ERBB2 was higher in APD compared to both PD and controls.

Since APD include both tauopathies (i.e., PSP and CBS) and a synucleinopathy (MSA) and to distinguish these individual diagnoses along PD (a synucleinopathy), we also performed analysis for tauopathies versus PD (Additional file 1: Fig. S5a), tauopathies versus synucleinopathies (Additional file 1: Fig. S5b), MSA versus PD (Additional file 1: Fig. S5c) and MSA versus controls (Additional file 1: Fig. S5d). For analyses of tauopathies versus PD and synucleinopathies versus tauopathies, the data were not adjusted for age and sex due to low number of cases. Many identified proteins overlapped with the APD versus PD analysis. However, this more detailed analysis revealed that Wnt inhibitory factor 1 (WIF-1) can distinguish tauopathies from PD and synucleinopathies. Moreover, the change of KLK10 found in the pooled APD group versus controls and PD was actually only present in the MSA patients (Additional file 1: Fig. S6). Both CBS and PSP showed no significant difference when compared with PD or controls.

### ELISA validation of CSF MK levels

Next, we performed a technical validation for MK, the most elevated protein in PD CSF from the PEA screening, using a commercially available ELISA kit with validated sensitivity [34, 35]. Data were first log2 converted to match the NPX values of the PEA. The ELISA results were similar to what we found with the PEA, but resulted in even more significant group differences (Fig. 4b, Additional file 1: Fig. S7). It also yielded an improved discriminatory performance between PD and controls, with an ROC AUC of 0.84 (data not shown). Furthermore, correlating individual values of the ELISA with those of the PEA showed a strong linear relationship, and z-scored concentrations highlight the similarity in inter-assay group comparisons.

Since MK is a cytokine, its change might suggest inflammatory processes. Therefore, we specifically examined if other cytokines in the assay (i.e., IL6, IL16, IL17D, IL18 and IL27) were also altered. Only IL17D was significantly increased in PD cases compared with controls in the Stockholm cohort (Fig. 1a), and it showed a trend of increase in the BioFIND cohort (*P* = 0.054). There was no correlation between MK and IL6 (Additional file 1: Fig. S8a) or IL16 (Additional file 1: Fig. S8b) levels. Results also showed weak correlations of MK with IL18 (Additional file 1: Fig. S8c) and IL27 (Additional file 1: Fig. S8d) and strong correlation of MK with IL17D (Additional file 1: Fig. S8e).

### Validation of PSP and CBD biomarkers in a second cohort

**Fig. 5:**
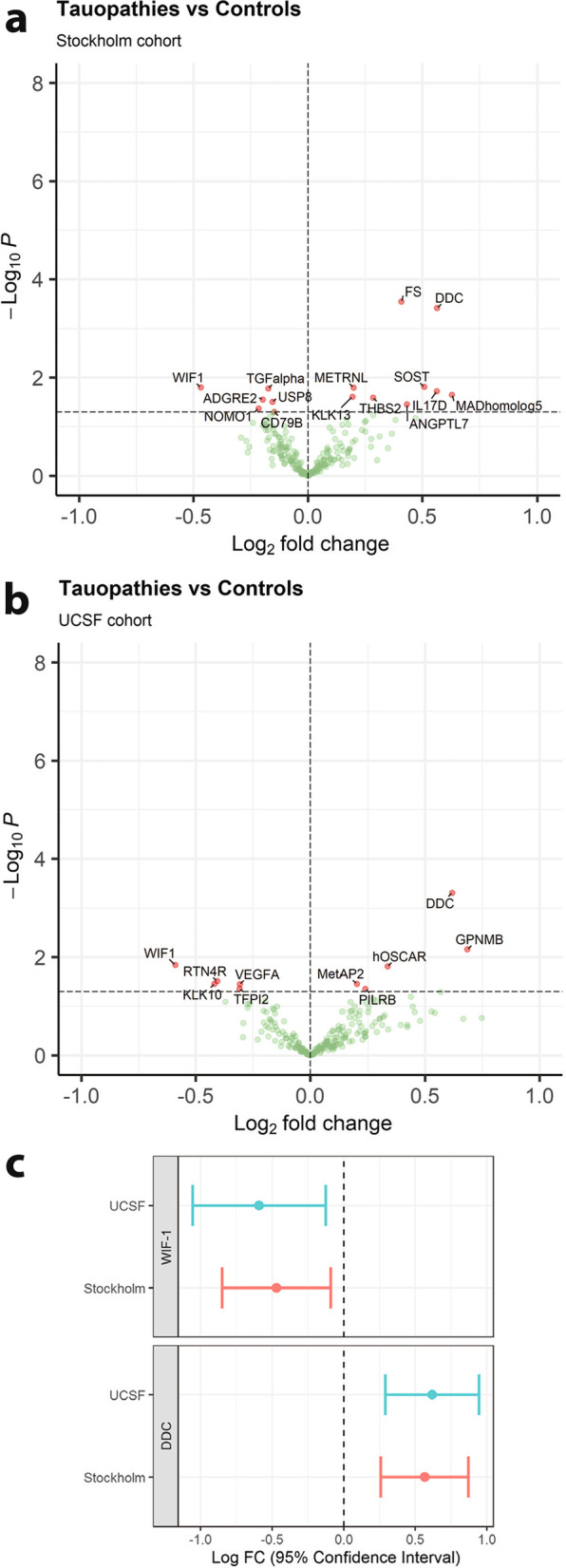
Altered CSF proteins between 4R-Tauopathies and Controls. Volcano plots showing log2 fold change and -log10 *P*-value for the Stockholm (**a**) and the UCSF cohorts (**b**), with a forest plot showing that the overlapping significant proteins WIF-1 and DDC had consistent significant changes in the UCSF cohort and the Stockholm cohort (**c**)

Lastly, we analysed CSF from individuals with pathologically confirmed PSP or CBD along with controls from the UCSF cohort, and results were compared to those from the Stockholm cohort. The PSP and CBD individuals were combined into the 4R-Tauopathy group to increase power. In total, 69 controls and 18 4R-Tauopathies were assessed in the Stockholm cohort, while 11 controls and 25 4R-Tauopathies were assessed in the UCSF cohort. The data were adjusted for age and sex, and volcano plots were compared between the two cohorts (Fig. 5). Due to the small size of the UCSF cohort, unadjusted *P*-values were used and overlapping differential proteins between the two cohorts were compared in a forest plot. The UCSF cohort and the Stockholm cohort showed consistent significant changes in WIF-1 and DDC (Fig. 5c).

## Discussion

In our study, we used a large-scale multiplex PEA approach to identify CSF markers in a cohort of PD, APD, and controls. Compared to previous PEA studies that implemented Neurology and Inflammation panels [15, 16], we used Oncology II, Cardiovascular II and Metabolism panels based on pilot data performed against 12 different panels.

In total, we identified 26 and 39 significantly changed proteins in PD compared with controls in Stockholm and BioFIND cohorts, respectively. These proteins are implicated in a number of different processes in the brain, e.g., neurogenesis, inflammation, iron balance and lysosomal protein degradation pathways, all implicated in PD pathology. Moreover, they are located in the extracellular region or are secreted, which strengthens their biomarker potential. They are most abundant in peripheral locations, except a few that are most highly expressed in the brain. Importantly, six proteins (MK, DDC, SMAD5, CCL17, TFPI-2 and TF) were changed in both cohorts. Biological function (UniProt database), cellular location (UniProt database) and tissue expression (GTEx database) of the above proteins are summarised in Additional file 1: Table S6.

The most significant protein, MK, was further validated by ELISA and displayed a good discriminatory performance between PD and controls (AUC 0.84). MK is a growth factor overexpressed in various pathologies [36], and has been extensively studied as a cancer biomarker [37]. As a soluble cytokine, it is released from cells and quickly apparent in body fluids such as blood, urine and CSF, making it a good biomarker for disease detection. MK has been observed in Aβ deposits in AD patients and in extracellular neurofibrillary tangles in patients with parkinsonism-dementia complex of Guam, and has been shown to inhibit Aβ fibril formation and cytotoxicity [38]. MK knockout mice exhibit delayed hippocampal development, and show impairment of olfactory discrimination and short-term social recognition memory, as well as decreased levels of tyrosine hydroxylase, dopamine and its receptors in the striatum [39, 40]. Therefore, they are suggested to be an early PD model. Since MK is a cytokine, it may reflect inflammatory processes. Here, we correlated the levels of MK with the other cytokines measured in the assay. We observed positive significant correlations with the anti-inflammatory cytokines IL-17D and IL-27. IL-17D in the brain is mainly expressed by astrocytes and has been suggested to play a role in local immune responses by inducing myeloid growth factors and chemokines [41]. IL-27 is expressed in microglia, astrocytes, and neurons [42]. In addition, MK had no or weak negative correlations with the pro-inflammatory cytokines IL-6, IL-16 and IL-18.

DDC, which was significantly increased in PD, catalyses the conversion of aromatic amino acids to the corresponding amines, including the central conversion of *L*-DOPA to dopamine in PD [43, 44]. Increased CSF and plasma levels of DDC in PD patients, both treated and untreated, are reported in a recent preprint [45]. Moreover, in agreement with our results, the CSF DDC levels are not associated with clinical motor scores. An insufficient DDC synthesis leads to impaired motor coordination, disturbance in cognitive and physiological homeostasis, many neuropsychiatric disorders and severe developmental delay [46]. We observed correlations of DDC level with LEDD and LDD in PD patients; however, DDC was also significantly increased in drug-naïve patients. Therefore, it might be also part of the compensatory mechanism to increase dopamine amount. It has been reported that *DDC* gene polymorphisms can affect the enzymatic activity of DDC [47] and consequently change the response to levodopa treatment. It has been shown that this modulation does not occur in the periphery, but rather in the brain, and the mechanism(s) of how *DDC* gene polymorphisms affect levodopa responsiveness are still largely unknown [48, 49]. It has long been known that converting levodopa to dopamine in the periphery is detrimental for treatment, since dopamine cannot pass the blood–brain barrier. However, it is possible that a higher amount of DDC in the brain, reflected by CSF DDC levels, can more efficiently convert levodopa to dopamine, providing faster response to the treatment. Still, further research will be necessary to elucidate how CSF DDC levels correspond to levodopa responsiveness in patients.

The increased CSF level of MAD homolog 5 in PD patients suggests a possible mechanism of increased iron deposits in PD patients [50]. MAD homolog 5 is a signalling molecule downstream of bone morphogenetic protein 6 (BMP6), which was also increased in the Stockholm cohort PD patients. Knockout of MAD homolog 5 reduces the total number of newly generated neurons and forces cells to exit cell cycle, leading to premature neurogenesis [51]. However, the role of MAD homolog 5 in neurodegeneration remains elusive. BMP6 is a member of the TGFβ superfamily and its deregulation is involved in cancer [52]. BMP6 expression is upregulated by increased iron, causing higher expression of hepcidin, which in turn limits iron absorption and recycling [53]. Interestingly, increased BMP6 has been detected in the brains of AD patients and APP transgenic mice, accompanied by impaired neurogenesis [54]. In addition, BMP6 has neurotrophic and neuroprotective activities [55]. These findings suggest that BMP6, in addition to its importance for iron homeostasis, plays a role in the protection against various toxins and in neuronal repair after neurodegeneration.

TFPI2 is a proteolytic enzyme inhibitor belonging to the superfamily of serine protease inhibitors. It inhibits the extracellular matrix hydrolysis via suppression of matrix metalloproteinases [56]. TF is a main target of TFPI2 [57]. Changes in TF and TFPI2 might suggest problems with blood coagulation [58, 59] and have no clear connection with brain or neurodegenerative disorders.

The focus of this study was to identify CSF biomarkers to aid in PD diagnosis, but we also made some findings related to APD. KLK10 is a serine protease engaged in various processes in the periphery [60] and in the brain [61]. In this study, KLK10 was decreased in APD CSF but unchanged in PD compared to controls. The change was especially high in MSA patients, indicating KLK10 as a potential biomarker to distinguish MSA from PD and control cases. The dysregulation of KLKs has been shown to contribute to many neurodegenerative and neurological disorders [62]. For instance, lower levels of KLK10 have been found in the CSF of frontotemporal dementia and AD patients [63]. In summary, the changes in the above-mentioned proteins in the CSF highlight inflammation as a contributing factor in neurodegenerative disorders.

Finally, WIF1 which was consistently decreased in PSP and CBS compared to controls in the two independent cohorts, is an inhibitor of Wnt/β-catenin signalling pathway and regulates processes like cell proliferation and differentiation [64]. It is closely associated with regenerative events in the brain and retina [65].

This study has some limitations. This study was focused on PD biomarkers, and consequently the studied three PEA panels were selected from a pilot study of 12 PEA panels using only PD and control samples. Therefore, we might have neglected PEA panels that are well suited for biomarkers of APD. Moreover, as the numbers of patients with the rare disorders CBS, PSP and MSA were relatively low, they were combined into one heterogeneous APD group in order to gain statistical power. This was fine when identifying markers specific for PD, but it would be of interest to see how the studied proteins differ between different APDs. Another consideration is that most PD patients in the study were recently diagnosed, and many were not yet on any medication. This is beneficial when looking for early diagnostic biomarkers, but the trade-off is that patients were at very similar disease stages, and had not yet developed many of the non-motor symptoms, hence more subtle biomarker correlations with symptom severity might have been missed.

## Conclusions

We show novel promising protein markers for PD and related disorders using PEA technology at a large scale. In addition to validating in two cohorts MK, DDC, SMAD5, CCL17, TFPI-2 and TF for PD, we also identified a few specific biomarkers that can differentiate between PD and APD. In addition, we validated PEA as a reliable multiplex technique by comparing the level of MK measured by PEA with that by the ELISA approach. Although we replicated the findings in two cohorts from two different sites, studies in other cohorts are needed to verify the findings.

### Supplementary Information

**Additional file 1**. **Fig. S1**. PD patient height vs CSF protein levels. **Fig. S2**. Association of aromatic *L*-amino-acid decarboxylase (DDC) levels with levodopa daily dose (LDD). **Fig. S3**. Effect of treatments other than levodopa on CSF proteins in the Stockholm cohort. **Fig. S4**. Effect of treatments other than levodopa on CSF proteins in the BioFIND cohort. **Fig. S5**. Altered CSF proteins in APD. **Fig. S6**. Levels of KLK10 in the Stockholm cohort patients. **Fig. S7**. Levels of MK in the Stockholm cohort patients. **Fig. S8**. Correlation of MK with IL6, IL16, IL18, IL27 and IL17D for all analysed cases (Spearman’s ρ) in both cohorts. **Table S1**. Stockholm cohort. **Table S2**. BioFIND cohort. **Table S3**. UCSF cohort. **Table S4**. Protein changes in confirmed and DaTSCAN-missing cases compared to controls. **Table S5**. Medications. **Table S6**. Significantly changed proteins in PD patients.

## Data Availability

The datasets used and/or analysed during the current study are available from the corresponding author upon reasonable request. BioFIND cohort data will be freely available at the BioFIND database (http://biofind.loni.usc.edu).

## Abbreviations

AD: Alzheimer’s disease
APD: Atypical parkinsonian disorders
AUC: Area under curve
CBD: Corticobasal degeneration
CBS: Corticobasal syndrome
CCL17: C–C motif chemokine 17
CSF: Cerebrospinal fluid
DDC: DOPA decarboxylase
DOPA: *L*-3,4-dihydroxyphenylalanine
ELISA: Enzyme-linked immunosorbent assay
IL17D: Interleukin-17D
KLK10: Kallikrein 10
LEDD: *L*-DOPA equivalent dose
LOD: Limit of detection
MAD: Mothers against decapentaplegic
MK: Midkine
MSA: Multiple system atrophy
NPX: Normalised protein expression
PD: Parkinson’s disease
PEA: Proximity extension assay
PSP: Progressive supranuclear palsy
ROC: Receiver operating characteristic
TF: Tissue factor
TFPI-2: Tissue factor pathway inhibitor 2
t-SNE: T-distributed Stochastic Neighbour Embedding
WIF-1: Wnt inhibitory factor 1
